# Dental students’ perception of objective structured clinical examination (OSCE): a cross-sectional study

**DOI:** 10.1038/s41405-026-00413-4

**Published:** 2026-03-17

**Authors:** Nissaf Daouahi, Sarra Nasri, Yosra Gassara, Rim Kallala, Jilani Saafi

**Affiliations:** 1 Department of Fixed Prosthodontics, Faculty of Dentistry, University of Monastir, Monastir, Tunisia; 2 Department of Fundamental Sciences, Faculty of Dentistry, University of Monastir, Monastir, Tunisia

**Keywords:** Health care, Dentistry

## Abstract

**Background:**

The Objective Structured Clinical Examination (OSCE) is increasingly used to assess clinical competence in dental education. Understanding students’ perceptions of this assessment method is essential for improving its design and educational impact, particularly in fixed prosthodontics.

**Objective:**

To assess dental students’ perceptions of OSCEs in Fixed Prosthodontics.

**Materials and methods:**

A cross-sectional study was conducted in May 2025 at the Department of Fixed Prosthodontics, Faculty of Dentistry, Tunisia. Final-year dental students (*n* = 144) who completed a fixed prosthodontics OSCE were invited to participate. Data were collected anonymously using a structured online questionnaire administered via Google Forms and QR code. The questionnaire assessed students’ perceptions of the OSCE in terms of clarity of instructions, organization, and stress level. Participation was voluntary.

**Results:**

A total of 144 final-year dental students completed the questionnaire. The response rate was 100%. The majority of respondents (87%) reported that the OSCE content and instructions were clearly explained. Similarly, 87.5% indicated that they felt comfortable with the examiners before the examination. Regarding time allocation, 36.1% of students reported a neutral perception, while 11.8% indicated that the time available at certain stations was insufficient. In terms of stress, 65.9% of respondents described the OSCE as moderately stressful. With respect to perceived difficulty, most students reported greater difficulty in the cognitive station (43.1%), followed by the communication and behavioural station (33.3%). Finally, 93% of respondents reported that the competencies assessed during the OSCE were aligned with the objectives of their clinical training.

**Conclusion:**

This study shows that students perceived the OSCE as an appropriate assessment method for clinical skills in fixed prosthodontics. They were generally satisfied with its clarity and organisation. However, they reported that some aspects should be improved, such as the timing and stress management.

## Introduction

A graduate dentist should have different skills, including investigation, problem-solving, communication, and practical skills. In the Tunisian dentistry school, the assessment of students’s competence has been for a long time limited to oral and written examinations, primarily in the form of multiple-choice questions (MCQs). Clinical performance used to use non-standardized and subjective criteria that can vary between assessors [1].

The Tunisian dental institutions have progressively developed other assessment strategies. It aimed at better simulating real clinical practice and enhancing the overall quality of dental education [2, 3].

The Objective Structured Clinical Examination (OSCE) has emerged as an innovative and relevant assessment strategy in healthcare education. It is now widely recognized as a reliable tool for evaluating clinical competence in a standardized environment [4]. It uses a series of structured stations with standardized clinical scenarios, predefined assessment criteria, and objective scoring systems. It often combines checklists with global rating scales [5, 6]. Contrary to written and oral examinations which focus on on Miller’s second level of competency ‘Know How’, OSCE targets the third level ‘Show How’ [7].

The perception of dental students regarding OSCEs is an important factor in evaluating their educational effectiveness. Contemporary evidence suggests that OSCEs may induce significant anxiety among dental students. A recent cross-sectional study reported that the majority of dental students experienced moderate to high anxiety during the OSCE [8].

According to the litterature, some studies have shown that many students perceive the OSCE as comprehensive tool of clinical skills evaluation; meanwhile, others are dealing with a stressful experience. Pierre et al. Confirm that OSCE sessions focus on strengths and weaknesses in clinical compétences [9]. Alkhatib et al. (2013) found that many students expressed concerns about time pressure and artificial aspects of the scenarios [10]. However, large-scale surveys indicate that a substantial proportion of students consistently perceive OSCEs as fair, well-structured, and comprehensive tools for assessing clinical skills when compared with traditional formats [8]. Moreover, recent mixed-method research also showed that over 65% of dental students reported positive contributions to competency development, particularly in technical proficiency and clinical decision-making [13]. Yet, this tool of evaluation presents limitations related to resources and equipments which make it financially expensive for institutes [11, 12].

This investigation aims to explore students’ perceptions of the OSCE in fixed prosthodontics clinical training at the Dentistry school of Tunisia. It includes aspects such as organisational instructions and stress management.

## Materials and methods

### Study design

The study was conducted at the Dentistry school in Tunisia at the end of the fifth year of study. This was a cross-sectional observational study using a survey design. It aimed to assess the dental students’ perception of the Objective Structured Clinical Examination (OSCE). The latter was structured into stations, each designed to assess specific competencies in fixed prosthodontics, including diagnostic treatment planning élaboration, technical procedures, and communication skills. Each station was supervised by a professor or assistant professor using a standardized scoring checklist.

### Study population

The study included 144 final-year dental students (five groups; 67 male/ 77 female) who had completed their final clinical training in the department of fixed prosthodontics. The evaluation was carried out in June 2025, during the week. All responses were anonymous. The participation in the survey was voluntary. The response rate was 100%.

### Questionnaire [9, 14]

The questionnaire was developed using Google Forms and QR codes. It includes Perception-Based Questions, and it is associated with Likert-scale items ranging from (1) Strongly Disagree to (5) Strongly Agree. It served as a feedback tool and assessed organizational and instructional quality, and stress level. The principal measures of this questionnaire were students’ perception of the examination aspects.

The questionnaire used in our research was not newly developed. It was adopted as a reference from a previously validated instrument. The original questionnaire was developed and validated by Pierre et al. (2004) to assess students’ perceptions of the Objective Structured Clinical Examination (OSCE). Only minor and carefully considered modifications were introduced to adapt it to the Tunisian context; specifically adjusted and simplified to enhance clarity and accessibility for students [9]

### Ethical considerations

The purpose of the study was discussed with the head of the department of fixed prosthodontics, and it was indicated that data would be analysed and presented anonymously. This study used an anonymous questionnaire-based educational evaluation with no patient involvement or identifiable personal data; therefore, formal ethical approval was not required in accordance with institutional guidelines.

This was an anonymous survey of dental students and completion of the questionnaire implied consent to participate

### Data collection and analysis

Data collection was carried out over one week following the final year evaluation. The responses were automatically recorded and exported from Google Forms into Microsoft Excel, then analysed using SPSS software. Descriptive statistics, including frequencies, percentages, means, and standard deviations were used to summarize the data. Differences in perceptions by gender or other variables were explored using Chi-square tests, with a significance level set at *p* < 0.05.

## Results

**Fig. 1:**
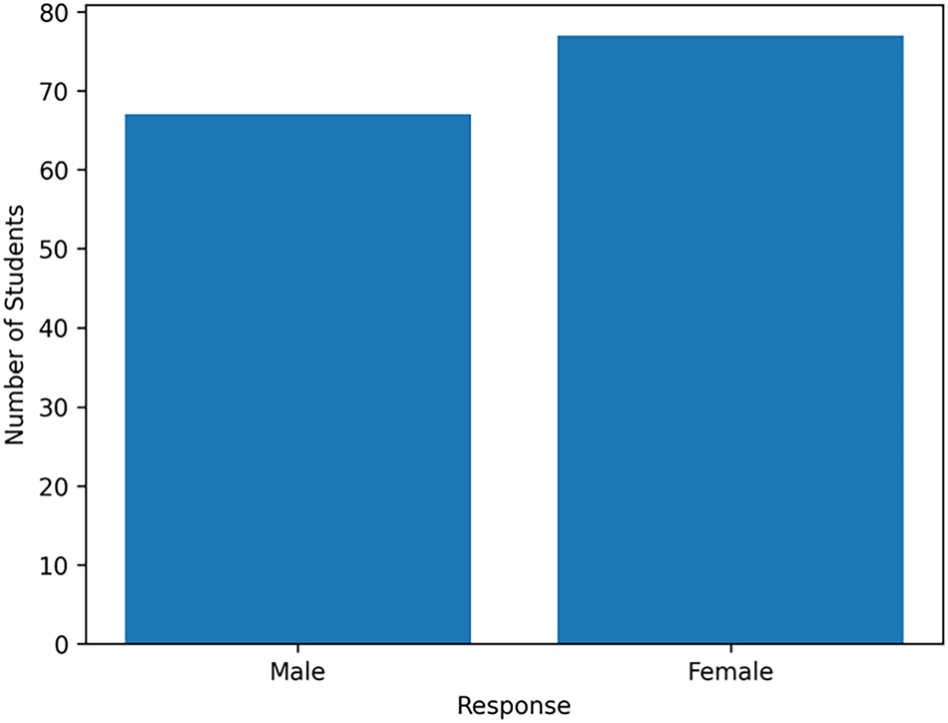
Gender distribution of students. This figure presents the proportion of male and female students included in the study. Each bar represents the percentage of students within each gender category.

From the 144 students, the response rate was 100%. Table 1 mentions the gender and the day of the OSCE session (Fig. 1).

**Table 1:** The gender and the day of the evaluation.

		Frequency	Percentage
The gender	Male	67	46.5
	Female	77	53.5
	Total	144	100
The day of the evaluation	Monday (26/5/2025)	32	22.2
	Tuesday (27/5/2025)	29	2.1
	Wednesday (28/5/2025)	31	21.5
	Thursday (29/5/2025)	26	18.1
	Friday (30/5/2025)	26	18.1

**Fig. 2:**
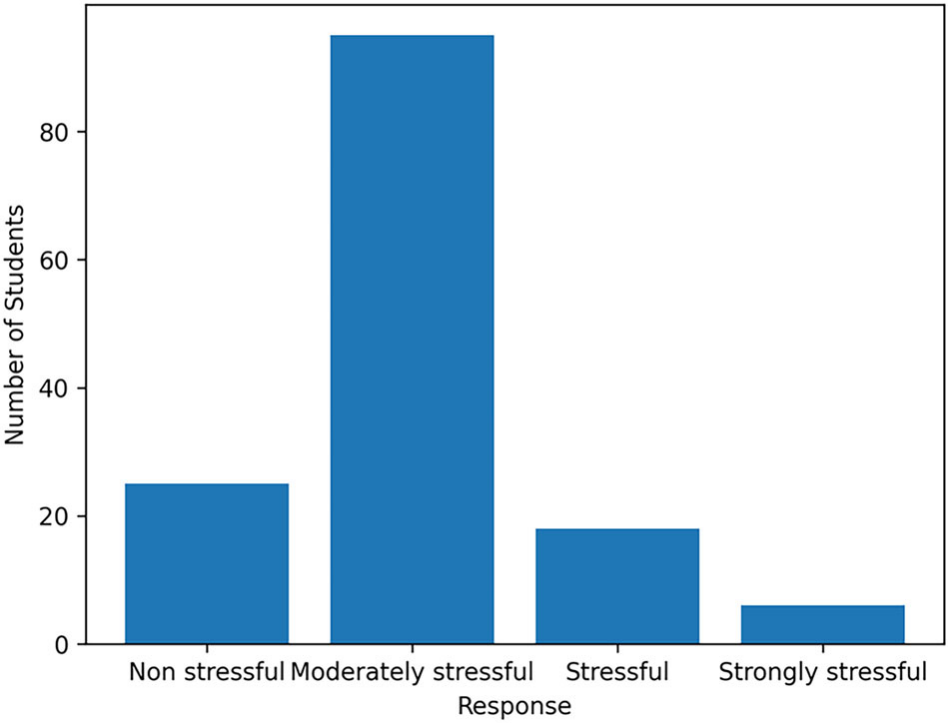
Stress level during OSCE. This figure illustrates the level of stress reported by students during the Objective Structured Clinical Examination (OSCE). Bars represent the proportion of students within each stress category.

**Fig. 3:**
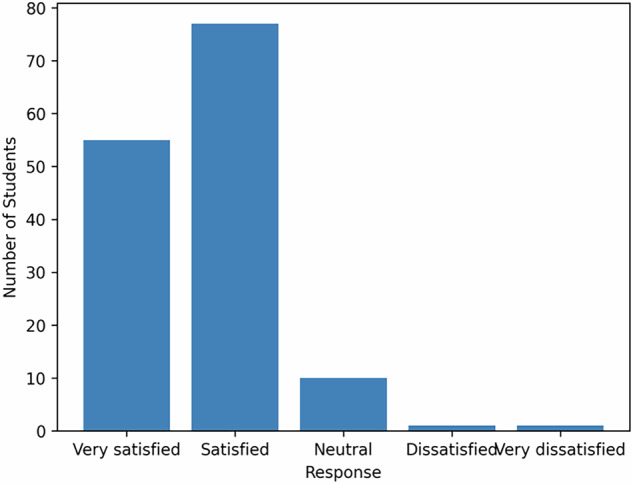
Student Satisfaction with OSCE. This figure displays students’ satisfaction levels regarding the OSCE as an assessment method.

**Fig. 4:**
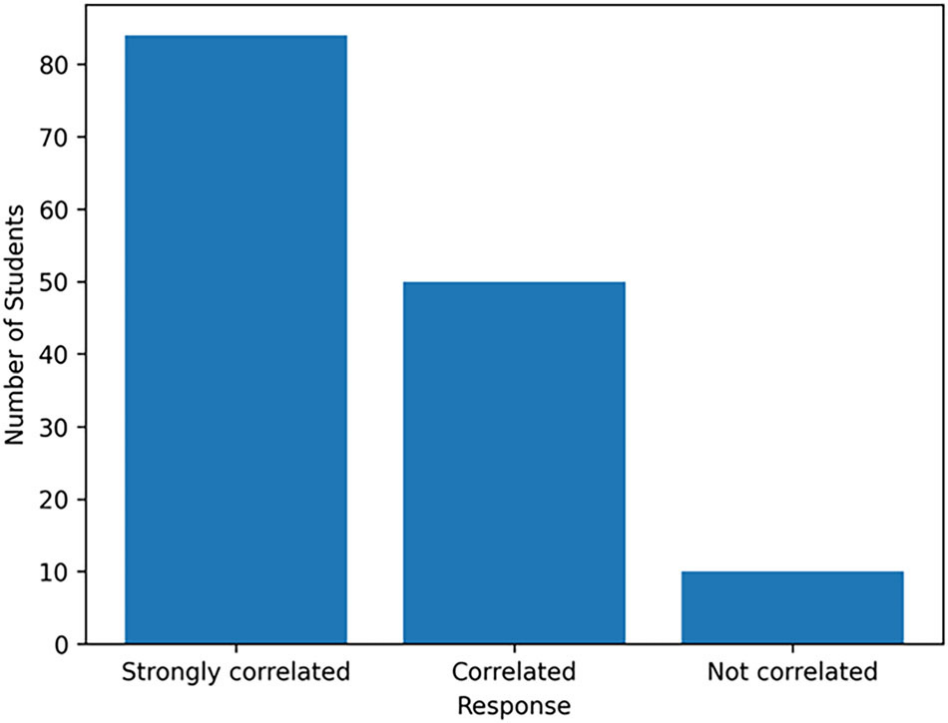
Alignment of OSCE with training objectives. This figure shows students’ perceptions of how well OSCE aligns with educational and clinical training objectives.

The results were presented through 3 tables (Tables 2, 3, and 4) where the frequency and the percentage for each response or attitude were mentioned. 91.7% were generally satisfied with the organizational aspects (Figs 2–4).

- 87% of students agreed or strongly agreed that the OSCE content and examiner instructions were clearly explained.
- 93% reported that the OSCE competencies matched the goals of their clinical training.
- 65.9% described the OSCE as “moderately stressful”, while only 4.2% found it “strongly stressful.”
- 11.8% suggested that time pressure is a potential area for improvement.

**Table 2:** The content, stress level, and attitude of professors.

	Response	Frequency	Percentage
1. Is the content of the evaluation well explained?	Strongly Agree	73	50.7
	Agree	52	36.1
	Neither agree or disagree	12	8.3
	Disagree	4	2.8
	Strongly Disagree	3	2.1
2. Did the examiners make you feel comfortable?	Strongly agree	74	51.4
	Agree	52	36.1
	Neither agree or disagree	14	9.7
	Disagree	3	2.1
	Strongly Disagree	1	O.7
3. Stress level	Moderately stressful	95	65.9
	Non stressful	25	17.4
	Stressful	18	12.5
	Strongly stressful	6	4.2

**Table 3:** Timing, clarity of instructions, and level of difficulty.

	Response	Frequency	Percentage
Is the timing sufficient?	Agree	60	41.7
	Neither agree nor disagree	52	36.1
	Strongly Agree	15	10.4
	Disagree	13	9.0
	Strongly Disagree	4	2.8
Are the instructions clear?	Agree	71	49.3
	Strongly Agree	54	37.5
	Neither agree nor disagree	16	11.5
	Disagree	2	1.4
	Strongly Disagree	1	0.7
Difficulties	Cognitive	62	43.1
	Communication skills	48	33.3
	Practical skills	34	23.6

**Table 4:** General satisfaction and correlation with the objectives of the training.

	Response	Frequency	Percentage
General satisfaction	satisfied	77	53.5
	Very satisfied	55	38.2
	Neither satisfied or dissatisfied	10	6.9
	Dissatisfied	1	0.7
	Very Dissatisfied	1	0.7
Correlation between the evaluation and the training	Strongly correlated	84	58.3
	Correlated	50	34.7
	Not correlated	10	6.9

### OSCE results—representation with diagrams

(Figures 1–4)

## Discussion

The Objective Structured Clinical Examination (OSCE) is an international assessment tool used to evaluate clinical competencies in medical schools [14] objectively. Compared to multiple-choice Questions MCQs, it is becoming more prevalent in health care education because it assesses practical skills [15]. It is widely recognised for its ability to identify deficiencies in clinical competencies. In addition, it has also been categorised by the American Dental Education Association Commission on Change and Innovation as an excellent format for evaluating a full range of competencies, especially those related to diagnosis and treatment [16]. In the UK, it is widely used as high-stakes summative exams that determine year progression and/or graduation in dental schools. Immediate or structured feedback, OSCEs are being introduced early in the curriculum to support learning as well as judgment of competence [17].

During COVID-19, several UK/European schools piloted virtual OSCEs (VOSCEs) over videoconference as an alternative clinical assessment, largely judged by staff and students to be a good alternative if well planned [18–22].

In Saudi Arabia, Eswi et al. (2013) confirm that more than half of the nursing students reported that MCQs are the easiest assessment format [22]. This can be related to the possibility of easily obtaining marks compared to the OSCE [14].

Dental OSCE implementation has been described in general dentistry, restorative, and periodontology contexts. In fixed prosthodontics, it remains under–reported in the literature. Few studies have specifically examined the use of structured clinical examinations for assessing competencies.

The present study addresses this important gap by investigating dental students’ perceptions of the OSCE in fixed prosthodontics within a Tunisian dental school. This represents a novel contribution to the global evidence base in this specialty, where students must integrate clinical skills, including teeth preparation, impressions, colour matching, and bonding protocols, with clinical cases presenting different degrees of complexity [23, 24].

Chambers (2000) and Schoonheim-Klein et al. (2008) demonstrated that OSCEs align more closely with real-world clinical performance compared to traditional exams [25, 26]. According to Yiu et al. (2011), students viewed that OSCE provides an authentic learning experience leading to the integration of knowledge in a realistic clinical context [27].

This cross-sectional study offers valuable insights into final-year dental students’ perceptions of the OSCE in fixed prosthodontics at the Tunisian dentistry school. The findings indicate positive student appreciation for clear communication and alignment with the objectives of the clinical training [28, 29]. This positive perception aligns with dental OSCE studies that report high satisfaction with organisation and standardized evaluation. This study provides discipline-specific evidence that OSCEs are a valid approach for assessing fixed prosthodontic competencies, particularly higher-order clinical reasoning in prosthesis design, which is insufficiently captured by conventional written or oral examinations [30].

Most students perceived the OSCE as moderately stressful (65.9%), with only a small proportion reporting high stress levels (4.2%). These findings indicate that, overall, the OSCE generated a manageable degree of anxiety for the majority of participants, although a subset of students may require additional psychological preparation. With regard to station timing, 11.8% of students reported that the allotted time was insufficient, suggesting time pressure as a potential target for refinement, whereas 36.1% expressed a neutral stance.

Kyong-Jee Kim, investigated, in 2016, the factors associated with medical students’ test anxiety during the OSCE. He found that students who likely feel more anxious about the OSCE may reduce their performance. He focused on enhancing the understanding of the various factors that influence test anxiety in OSCEs and developing effective educational interventions for helping students cope with such stress [31, 32].

A major finding is that approximately 87% reported that the OSCE content and professors’ instructions were clearly explained. This clarity is critical in reducing anxiety and enabling students to optimally perform during the evaluation. This reflects a good level of transparency in how the assessment was introduced.

OSCEs offer a standardized approach to assessment in dental education. It minimizes inter-student discrepancies and reduces examiner subjectivity.

In the present study, 75.7% of students agreed or strongly agreed that the assessment was standard across different groups, indicating effective standardization and supporting the objectivity of the OSCE format [33].

Most students reported difficulties in the cognitive station (43.1%), followed by communication and behaviour (33.3%). This can explain why clinical reasoning remains a challenging task. It means that Fixed prosthodontics requires advanced clinical reasoning, including interpretation of diagnostic data, treatment planning, and effective communication with patients [34].

The students appreciated the clarity of instructions and content, and the correlation with their clinical training. This confirms the authenticity of the evaluation. Stress and time constraints were noted as typical in high clinical assessments and should be improved in the future.

91.7% were generally satisfied with the organization, indicating good logistical and administrative planning, and 93% confirmed that the OSCE competencies matched the goals of their clinical training. All Chi-square tests were significant (*p* = 0), indicating that students’ responses were not due to random variation. There is still a need for more investigations regarding the effectiveness of OSCE in fixed prosthodontics [35].

Recent innovations in prosthodontic assessment include electronic OSCEs (e-OSCEs) designed for fixed prosthodontic tasks, such as fixed partial dentures, and implant-related competencies. These digitally supported formats have demonstrated stable student performance over time and improved standardisation and reliability of assessment. E-OSCEs represent a feasible and scalable approach that aligns with accreditation standards and may be readily adapted to different institutional contexts [36].

### Limitations and recommendations

The high response rate adds strength to the findings. However, the lack of qualitative data limits insight into students’ expériences. As the study was conducted at one institution, the findings may not be widely generalised. In addition, the absence of questionnaire piloting represents a methodological limitation in this study.

Future studies should incorporate pilot-tested instruments, qualitative methodologies, and multicentre designs to enhance methodological rigour. Early integration of OSCEs into clinical training, supported by digital learning tools and mock OSCEs, may improve student preparedness and reduce examination-related stress.

## Conclusion

This study shows that students perceived the OSCE as an appropriate assessment method for clinical skills in fixed prosthodontics. They were generally satisfied with its clarity and organisation. However, they reported that some aspects should be improved.

Positive student perceptions appear to reflect adequate organisation and management of the evaluation. However, improvements are needed in station timing. Strategies to reduce examination-related stress are highly recommended.

Multi-institutional comparative studies will, particularly, strengthen the current evidence base; It also contributes to international efforts to harmonise competency-based assessment within undergraduate dental education.

## Data Availability

The datasets generated and/or analysed during the current study are available from the corresponding author on reasonable request.
